# Reversible Data Hiding in JPEG Images Using Quantized DC

**DOI:** 10.3390/e21090835

**Published:** 2019-08-26

**Authors:** Suah Kim, Fangjun Huang, Hyoung Joong Kim

**Affiliations:** 1 School of Information Security, Korea University, Seoul 02821, Korea; 2 School of Data and Computer Science, Sun Yat-sen University, Guangzhou 510006, China

**Keywords:** reversible data hiding, JPEG, DCT coefficients, DC coefficients, entropy

## Abstract

Reversible data hiding in JPEG images has become an important topic due to the prevalence and overwhelming support of the JPEG image format these days. Much of the existing work focuses on embedding using AC (quantized alternating current coefficients) to maximize the embedding capacity while minimizing the distortion and the file size increase. Traditionally, DC (quantized direct current coefficients) are not used for embedding, due to the assumption that the embedding in DCs cause more distortion than embedding in ACs. However, for data analytic which extracts fine details as a feature, distortion in ACs is not acceptable, because they represent the fine details of the image. In this paper, we propose a novel reversible data hiding method which efficiently embeds in the DC. The propose method uses a novel DC prediction method to decrease the entropy of the prediction error histogram. The embedded image has higher PSNR, embedding capacity, and smaller file size increase. Furthermore, proposed method preserves all the fine details of the image.

## 1. Introduction

JPEG image file format has proved its dominance year after year. Even with the new image standard that supports higher efficiency and compression, it still comes as a default image standard for storing images in smartphones and computers. With unparalleled support for it in variety of devices and software, reversible data hiding JPEG image has become an important topic.

Reversible data hiding is an interoperable data hiding method, which preserves the original file format and has ability to recover the original image from the embedded or watermarked image. But it is different than robust watermarking scheme such as one proposed by Liu et al. [1], which focus on recovery of the embedded message under image processing attacks.

The embedded image should be as close as possible to the original image and the PSNR value is measured against different payload size to compare the performance of the data hiding capability.

Much of the research focused on reversible data hiding is in pixel domain. They are based on lossless compression [2,3,4,5,6], difference expansion [7,8,9,10,11,12,13], or histogram shifting [7,14,15,16,17,18,19,20,21,22,23,24,25,26,27,28,29,30,31,32,33,34,35,36,37,38,39]. A recent advances [19,20,39,40] use two dimensional histogram shifting to achieve higher embedding capacity and lower distortion. Furthermore, a new research field using reversible data hiding, which focuses on adapting reversible data hiding technique as an image enhancement technique, has emerged [41,42,43,44,45,46,47,48]. In particular, some [41,42] proposed using reversible data hiding technique for automatic image enhancement and use the reversibility as an advantage to save storage space. Reversible data hiding can be also used before image encryption [49,50] to hide additional data or to obscure the size of the image in a reversible way.

On the other hand, reversible data hiding in JPEG has not been extensively researched. There are three main approaches: the first approach modifies the quantization table to artificially increase the quantized DCT coefficients and embed [51,52]. Although this approach has high embedding capacity, the file size increase is much larger than the obtained embedding capacity. The second approach modifies the Huffman table [53,54,55]. Although this approach preserves the file size, the embedding capacity is quite limited. The third approach modifies the quantized DCT coefficients for embedding [56,57,58,59,60,61,62,63,64]. It is most logical approach given that it directly modifies the visual features without modifying other image parameters (such as Huffman table and quantization table).

The proposed method is a third approach, which uses a prediction scheme for quantized DC coefficients. The proposed prediction provide improved accuracy, which decreases the entropy of the quantized DC prediction error histogram to have large embedding capacity with lower distortion. Furthermore, proposed method only modifies the quantized DC values.

**Figure 1:**
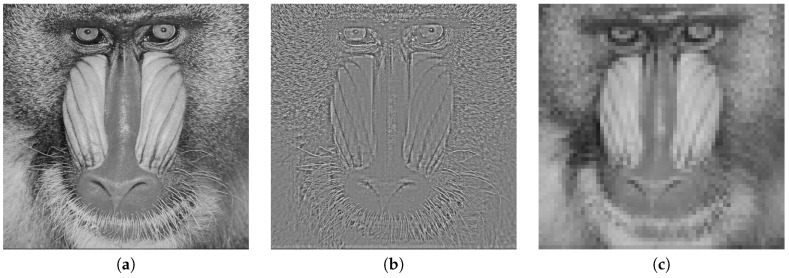
Reconstructed JPEG image of Baboon using different sets of quantized DCT coefficients. Quantized DC coefficients preserves the overall intensity, whereas quantized AC coefficients preserve the fine details of the image, making them less ideal for embedding for cases where fine details matter. (**a**) all quantized DCT coefficients; (**b**) quantized AC coefficients; (**c**) quantized DC coefficients.

The main goal of reversible data hiding in JPEG is to achieve high PSNR without causing much change in the file size, but there are specific cases where quantized AC coefficient preservation is required and is more important than achieving high PSNR. Consider a case where embedded images are used for image data analysis for searching specific patterns or an object. Image data analysis rely on feature extraction of fine image details, which the AC coefficients represent (see Figure 1 for an example). Without the AC coefficient preservation, data analytic may not perform as well.

The proposed method provides a novel reversible data hiding technique which can embed in the quantized DC coefficients. Unlike existing work, all the fine details are preserved since none of the AC coefficients are modified.

## 2. Brief Introduction to JPEG Baseline Encoding and Decoding

This section briefly describes the JPEG baseline encoding and decoding steps to aid with understanding of the proposed method. JPEG is a block based lossy compression technique which transforms 8 by 8 pixel blocks into to 8 by 8 quantized DCT coefficient blocks. The transformation consists of normalization by subtracting 128 from each of the pixels, discrete cosine transform (DCT), division by the quantization table, which is usually scaled using a scaling factor called quantization factor (QF) to control the effect of the compression and the image quality, and rounding is applied to make the values integral.

DCT is defined as following:

(1) $$DCT_{u,v}=\frac{\alpha (u)\alpha (v)}{4}\sum_{i=0}^{7}\sum_{j=0}^{7}p_{i,j}cos\left[\frac{(2i+1)u\pi }{16}\right]cos\left[\frac{(2j+1)v\pi }{16}\right]$$

where

(2) $$\alpha \left(u\right)=\left\{\begin{array}{cc} \frac{1}{\sqrt{2}} & \;\mathrm{if}\;u=0\\ 1 & \;\mathrm{if}\;else \end{array}\right.$$

where $p_{i,j}$ represents the pixel value at position (i,j) and $DCT_{u,v}$ represents the DCT value at position (u,v).

Once the quantized DCT coefficients are obtained, they are compressed losslessly. Quantized DCT coefficients consist of quantized DC and quantized AC coefficients. The quantized DC coefficient is the first DCT coefficient value and represents a form of a mean pixel value. The quantized AC coefficients are the rest of the DCT coefficients and represents the fine details of the pixel block. Differential pulse code modulation (DPCM), where the difference between two consecutive values is encoded, is used to compress the quantized DC coefficients in two adjacent blocks. Then, DPCM values are losslessly compressed using a variant of Huffman coding. For the quantized AC coefficients, run length coding is applied, and then encoded using a variant of Huffman coding.

To reconstruct the pixels from the quantized DCT coefficients, the quantized DCT coefficients are multiplied with the quantization table and then, inverse DCT is applied. Then, 128 is added to each value to undo the normalization, and finally, rounding function is used to make the result integral. More information about JPEG encoding and decoding can be found in the ISO document [65].

From here on, the quantized DCT coefficients (including quantized AC and DC coefficients) are denoted by bold font: DCT, AC, and DC.

## 3. Proposed Method

The proposed method uses a reversible data hiding technique called histogram shifting, where the performance is highly dependent on the prediction accuracy. The embedding and distortion of histogram shifting is relative to the prediction error; smaller prediction errors mean higher embedding capacity with lower distortion.

Previous work does not utilize advanced prediction in JPEG reversible data hiding. This is because of the assumption that the prediction in the DCT domain is difficult and is not worth the effort. Although the assumption is true in general, it is not true for all DCT. The first DCT in the DCT transformed block, also referred as the DC, represents the $\frac{8}{Q_{1}}\times$ mean normalized pixel values in the block, where $Q_{1}$ is the quantization table entry for quantizing the DC value. The proposed method builds upon this idea to propose a DC prediction method, which will increase the embedding capacity and decrease the distortion.

**Figure 2:**
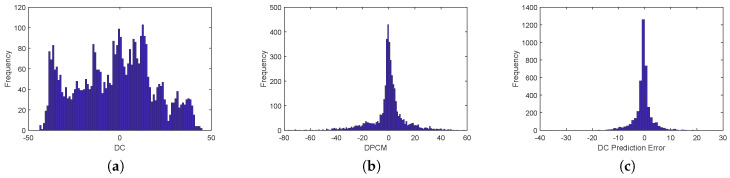
Comparison of different histograms. The proposed DC prediction error histogram has the highest peak and the smallest entropy. (**a**) DC histogram; (**b**) DPCM DC histogram; (**c**) DC prediction error histogram.

Figure 2 shows histograms of image Lena (QF = 50). The histogram of DCdoes not have a peak and has the largest entropy, the histogram of differential pulse code modulated (DPCM) DC is better for histogram shifting then DC histogram, because it has a high peak around 0 and lower entropy, and finally, the histogram of proposed DC prediction error histogram has the highest peak and the smallest entropy, making it ideal for histogram shifting.

The next subsections describe DC prediction method, embedding method, and extraction method.

### 3.1. DC Prediction

**Figure 3:**
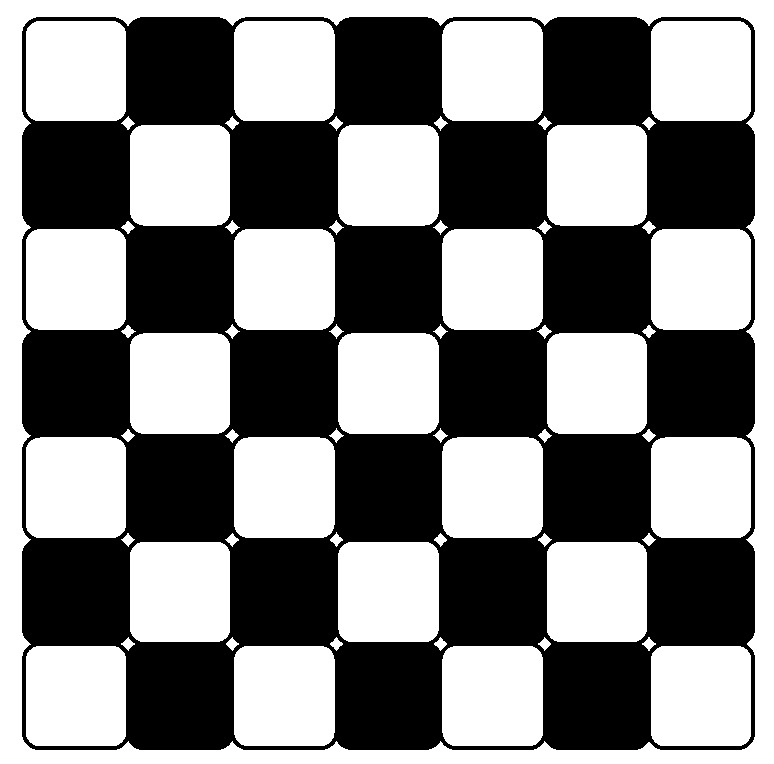
DCT blocks are divided in two alternating sets of white and black blocks.

Unlike the prediction in image compression, where only decoded blocks can be used for prediction, prediction in reversible data hiding can utilized all blocks which are not used for embedding. In other words, we can divide the blocks in to two non-overlapping alternating sets, “white” and “black” sets, and embed them one at a time to facilitate more accurate prediction (see Figure 3). Without loss of generality, the white set is embedded first then the black.

Before explaining the proposed prediction, we define ‘*T*’ or ‘target block’ as the block which we want to predict the DC of. Furthermore, the superscript ${}^{AC}$ is used to denote that the block is reconstructed only using the AC component.

Then, let $T^{AC}$ denote the partially reconstructed pixel target block using only the AC from the target block (DCvalue is set as 0). Then, because DCT is a linear function, *T* can be approximately decomposed as following:

(3) $$T\approx T^{AC}+DC\times \frac{Q_{1}}{8}$$

where $Q_{1}$ is the quantization value which divided the DC coefficient to get DC, and $\mathrm{DC}\times \frac{Q_{1}}{8}$ represents the effect of the inverse DCT on DC.

The proposed prediction uses the neighboring blocks and the partially reconstructed blocks using only the AC component. The neighboring DCT blocks are first transformed to pixel blocks of “North”, “East”, “South”, and “West”.

**Figure 4:**
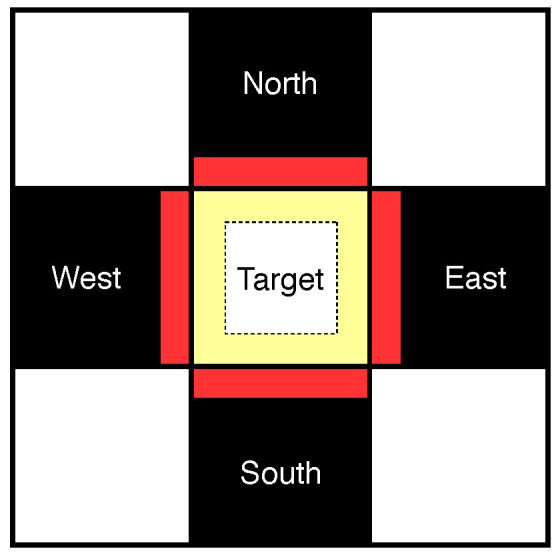
Example of the context used for prediction. DC is predicted using four direct neighboring black blocks and the AC of the target block.

**Figure 5:**
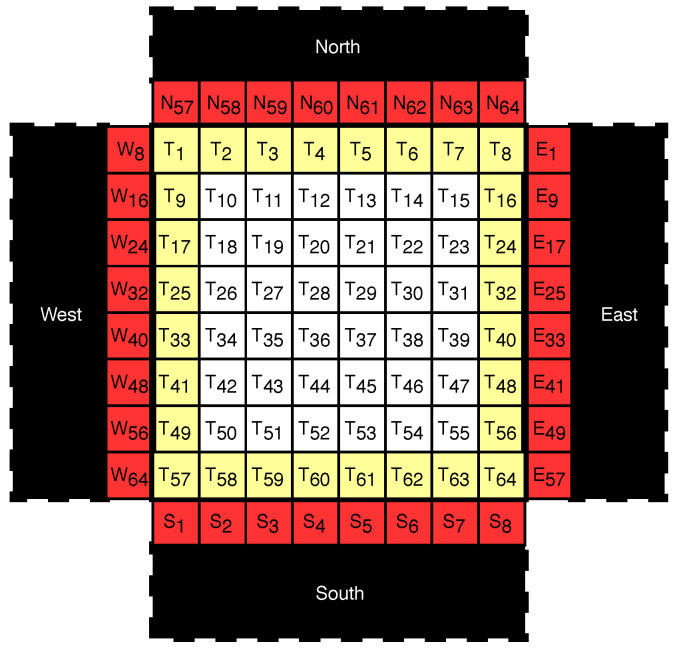
Contexts used for predicting DC. The neighboring pixel values from already decoded blocks (North, West, South, and East), and partially reconstructed target block $T^{AC}$ is used to improve the prediction accuracy.

Figure 4 shows graphical view of the division. The red area represents the parts of neighbors which are closest to the target block, and the yellow area represents the parts of the target block which are closest to the neighboring blocks. Using this setup, we assume that the pixels from the neighboring blocks and the pixels from the target block, which are exactly one position away from each other, should be similarly valued:

(4) $${\mathrm{Pixel}}_{\mathrm{red}}\approx {\mathrm{Pixel}}_{\mathrm{yellow}}$$

where ${\mathrm{Pixel}}_{\mathrm{red}}$, the pixel from the neighboring block, and ${\mathrm{Pixel}}_{\mathrm{yellow}}$, the pixel from the target block, are exactly one position away from each other. In Figure 5, $W_{8}$ is exactly one position away from $T_{1}$, but is two positions away from $N_{57}$ and $T_{9}$.

Combining Equations (3) and (4), we get following approximation:

(5) $${\mathrm{Pixel}}_{\mathrm{red}}\approx {\mathrm{Pixel}}_{\mathrm{yellow}}^{AC}+\mathrm{DC}\times \frac{Q_{1}}{8}$$

By rearranging the above equation, $\hat{\mathrm{DC}}$, the predicted value of DC is:

(6) $$\hat{\mathrm{DC}}=\left[\frac{8}{Q_{1}}\times \left({\mathrm{Pixel}}_{\mathrm{red}}-{\mathrm{Pixel}}_{\mathrm{yellow}}^{AC}\right)\right]$$

where [.] is the rounding function.

Finally, since there are multiple ${\mathrm{Pixel}}_{\mathrm{red}}$ and ${\mathrm{Pixel}}_{\mathrm{yellow}}^{AC}$, the mean is used as an estimator to evaluate $\hat{\mathrm{DC}}$:

(7) $$\begin{array}{cc} \hat{\mathrm{DC}} & =\left[\frac{8}{Q_{1}}\times \frac{\sum_{\mathrm{neighbor}}\left({\mathrm{Pixel}}_{\mathrm{red}}-{\mathrm{Pixel}}_{\mathrm{yellow}}^{AC}\right)}{\#\mathrm{of}\mathrm{neighbors}}\right] \end{array}$$

(8) $$\begin{array}{cc} & =\left[\frac{8}{Q_{1}}\times \frac{\sum_{\mathrm{North},\;\mathrm{East},\;\mathrm{South},\;\mathrm{West}}\left({\mathrm{Pixel}}_{\mathrm{red}}-{\mathrm{Pixel}}_{\mathrm{yellow}}^{AC}\right)}{32}\right] \end{array}$$

(9) $$\begin{array}{cc} & =\left[\frac{8}{Q_{1}}\times \frac{\sum_{n=1}^{8}\left(N_{56+n}-T_{n}^{AC}\right)+\left(E_{8n-7}-T_{8n}^{AC}\right)+\left(S_{n}-T_{n+56}^{AC}\right)+\left(W_{8n}-T_{8n-7}^{AC}\right)}{32}\right] \end{array}$$

### 3.2. Embedding

Histogram shifting technique is used to embed in the prediction error values ($\mathrm{DC}-\hat{\mathrm{DC}}$). Embedded DC is denoted by ${\mathrm{DC}}^{\prime }$, and is obtained using following equation:

(10) $${\mathrm{DC}}^{\prime }=\left\{\begin{array}{cc} \mathrm{DC}-b & \;\mathrm{if}\;\mathrm{DC}-\hat{\mathrm{DC}}=-1\\ \mathrm{DC}+b & \;\mathrm{if}\;\mathrm{DC}-\hat{\mathrm{DC}}=0\\ \mathrm{DC}-1 & \;\mathrm{if}\;\mathrm{DC}-\hat{\mathrm{DC}}<-1\\ \mathrm{DC}+1 & \;\mathrm{if}\;\mathrm{DC}-\hat{\mathrm{DC}}>0\\ \mathrm{DC} & \;\mathrm{else}\; \end{array}\right.$$

b∈{0,1} is the payload bit.

The histogram shifting technique shifts DC with prediction errors less than −1 by −1, so that DCs valued −1 can be embedded using coefficients valued −1 and −2: the coefficient valued −1 is modified to −2 if the payload bit is “1”, and left as −1 if the payload bit is “0”. Similar logic applies to DC with prediction error greater than 0. Since the histogram shifting is applied such that none are overlapping, it can be reversed. The next subsection discusses the extraction of the payload and the recovery of the original DC.

### 3.3. Extraction and Recovery

The extraction of the payload and the recovery of the original DC are trivial.

(11) $$b=\left\{\begin{array}{cc} 0 & \;\mathrm{if}\;{\mathrm{DC}}^{\prime }=-1\\ 0 & \;\mathrm{if}\;{\mathrm{DC}}^{\prime }=0\\ 1 & \;\mathrm{if}\;{\mathrm{DC}}^{\prime }=-2\\ 1 & \;\mathrm{if}\;{\mathrm{DC}}^{\prime }=1 \end{array}\right.$$

(12) $$\mathrm{DC}=\left\{\begin{array}{cc} {\mathrm{DC}}^{\prime }+1 & \;\mathrm{if}\;{\mathrm{DC}}^{\prime }-\hat{\mathrm{DC}}<-1\\ {\mathrm{DC}}^{\prime }-1 & \;\mathrm{if}\;{\mathrm{DC}}^{\prime }-\hat{\mathrm{DC}}>0\\ {\mathrm{DC}}^{\prime } & \;\mathrm{else}\; \end{array}\right.$$

### 3.4. Block Selection

Block selection is important for cases where full embedding capacity is not utilized. In order to have the smallest impact on the PSNR, blocks are sorted by their smoothness and blocks are embedded sequentially only until all the payload is embedded.

To ensure that the smoothest blocks are embedded first, block selection algorithm proposed by Huang et al. [62] is used. In their algorithm, number of zero DCT coefficients are used as a smoothness measure to sort the blocks. The assumption here is that the DCT blocks with more zero DCT coefficients will have less details and thus be smoother.

In the proposed method, zero DC coefficients are not used to measure the smoothness and only the zero AC coefficients are used to measure the smoothness. DC coefficients are not used, because they may change after embedding.

## 4. Encoder and Decoder

This section summarizes the encoding and decoding method of the proposed reversible data hiding scheme. Each subsection will describe the implementation steps and include minor implementation details to aid with understanding.

### 4.1. Encoder

1. Extract the DCT blocks from JPEG image.
2. Divide the blocks into white and black set.
3. Use block selection method to sort the white set of DC.
4. Predict the white set of DC and embed half of the payload.
5. Use block selection method to sort the black set of DC.
6. Predict the black set of DC and embed the rest of the payload including the payload length, which is appended in front of the rest of the payload. (Prediction uses the embedded ${\mathrm{DC}}^{\prime }$ from step 4.)

### 4.2. Decoder

1. Extract the embedded DCT blocks from JPEG image.
2. Divide the blocks into white and black set.
3. Use block selection method to sort the black set of ${\mathrm{DC}}^{\prime }$.
4. Predict the black set of ${\mathrm{DC}}^{\prime }$, extract the payload length and the half of the payload, and recover the original DC for the black set.
5. Use block selection method to sort the white set of DC.
6. Predict the white set of ${\mathrm{DC}}^{\prime }$, extract the first half of the payload, and recover the original DC for the white set. (Prediction uses the original DC recovered from step 4.)

## 5. Experiment

The performance of the proposed method is verified using PSNR (between the original JPEG and the embedded JPEG) and the file size gain (due to embedding) comparison. However, there are no existing work that focus only on embedding in DC. Thus, a variant of Huang et al.’s method [62], which is the state of the art algorithm in terms of PSNR and file size gain, is compared with the proposed method.

Methods such as Wang et al. [66], Xuan et al. [58], and Sakai et al. [59] are not compared here, as Huang et al.’s method produces images with higher PSNR with respect to the file size gain. See reference [62] for more detailed comparison. Improved version of Huang et al.’s work by Wedaj et al. [64] is not compared here either, since their method is the optimization of choosing multiple AC coefficients per block for embedding (the proposed method uses single DCT for embedding, comparison would not be fair). Without the optimization, Wedaj et al.’s method is the same as Huang et al.’s.

For fair comparison, a variant of Huang et al.’s method is used for the comparison. Although the original method only embeds in **AC**, it can be modified to embed in DPCM of DC with values 0 and −1. When embedded this way, Huang et al.’s method also preserves the **AC** coefficients just like the proposed method.

Quantization factor (QF) values of 50 and 80 are chosen for comparison. QF =50 based quantization table is the recommended base quantization table written in the standard document, which is scaled to obtain other quantization table, making it a good benchmark for test against. QF =80 based quantization table is the table which is known to achieve good compression to visual degradation ratio.

The code for the proposed method will be available at https://github.com/suahnkim/jpegrwdc (accessed on 23 August 2019).

## 6. Discussion and Analysis

For testing, two image sets are chosen. To see the comparison using the well-known images, the first image set includes 6 images, “Lena”, “Boat”, “Barbara”, “Baboon”, “Peppers”, and “F16” from USC-SIPI image data set (http://sipi.usc.edu/database/) (accessed on 23 August 2019). The original bmp images are JPEG compressed to the size of 512 × 512. These images are chosen specifically for comparing specific unique features. For more general comparison, the second image set includes 1000 images from Alaska image data set (https://alaska.utt.fr/) (accessed on 23 August 2019). The original raw .cr2 images are JPEG compressed to the size of 432 × 648.

### 6.1. Comparison Using USC-SIPI Image Data Set

In this section, USC-SIPI database is used to compare for specific features. PSNR value relative to the size of the embedded payload and the file size gain due to embedding the payload are compared.

#### 6.1.1. PSNR Comparison Using USC-SIPI Image Data Set

**Figure 6:**
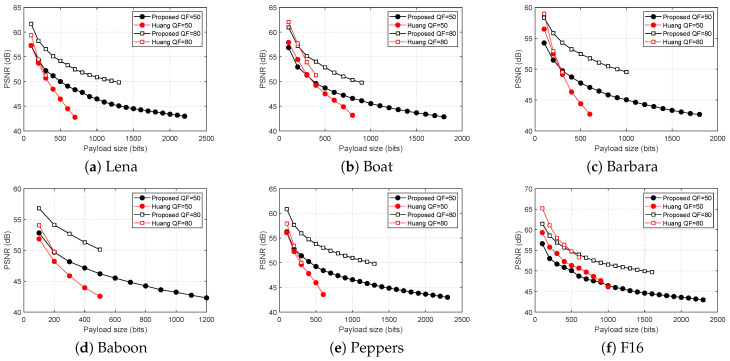
PSNR comparison for different payload sizes.

Figure 6 summarizes the PSNR comparison: “Huang QF = 50” represents the result when Huang et al.’s method is used to embed in the QF = 50 image, and “Huang QF = 80” represents the result when Huang et al.’s method is used to embed in the QF = 80 image. Same notation applies for the proposed method.

Both methods preserve the AC coefficients as required, but the proposed method has higher PSNR and embedding capacity for almost all images except the image F16. The proposed method consistently can embed more than 15% of the total blocks, and for few of the images it can embed more than 50%, whereas Huang et al.’s method can only embed between 5% and 25%.

Upon close inspection on the prediction error histograms of F16, DPCM is a better predictor for first few sets of payload. This is not surprising because F16 is a very smooth image. However, this is a single outlier, and the proposed method’s prediction is generally better and more consistently accurate than DPCM. More importantly, the proposed method can consistently embed more than Huang et al.’s.

#### 6.1.2. File Size Gain Comparison Using USC-SIPI Image Data Set

File size gain due to embedding is an important measure in JPEG reversible data hiding. JPEG is designed to offer good compression capability with respect to the image quality, thus reversible data hiding should not increase the file size significantly. Ideally, the file size gain due to embedding should be smaller than the embedded payload size.

**Figure 7:**
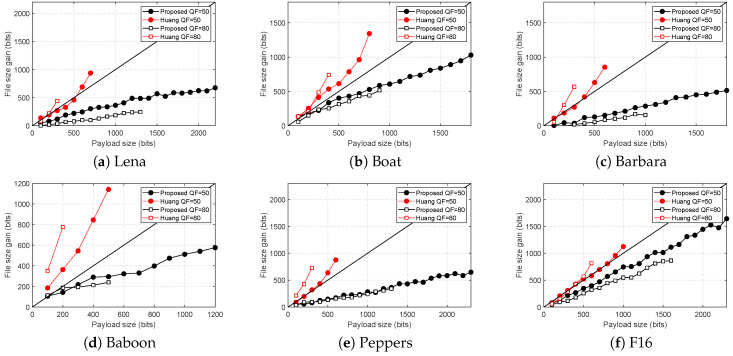
File size comparison for different payload sizes. The black straight line in the middle represents the equality for the file size gain and payload sizes.

Figure 7 summarizes the comparison result for the file size gain. The file size gain is measured against the size of the embedded payload. To clearly see whether the embedding caused more file size gain than the size of the payload, a straight line through the middle is drawn. The proposed method consistently has smaller file size gain than Huang et al.’s for both QFs. Furthermore, it has smaller file size gain than the size of the payload for all cases. This implies that when proposed method is used for embedding, it will not increase the file size more than the size of the payload.

### 6.2. Comparison Using Alaska Image Data Set

In this section, performance comparison using Alaska image data set is summarized. Unlike the earlier comparison which compared specific images, the comparison here uses 1000 images to compare using more robust statistical results. For the comparison, PSNR gain, file size difference, and payload gain are compared.

Applying reversible data hiding in 1000 JPEG images produces many data points: each image has different maximum payload size, and each payload size gives different PSNR value and file size. Fair comparison is done by only comparing the results for the same image and same payload size.

#### 6.2.1. PSNR Gain Comparison Using Alaska Image Data Set

**Figure 8:**
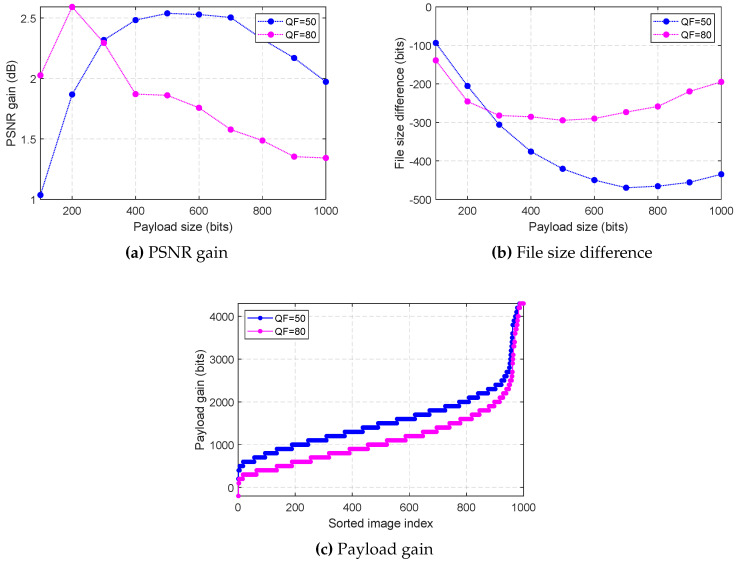
Extended comparison using Alaska image data set.

PSNR gain measures the difference between the PSNR values of the embedded image using proposed method and Huang et al.’s. Figure 8a summarizes the result for the average PSNR difference for different payload sizes. The results are all positive, meaning that the proposed method produced embedded images with higher PSNR than Huang et al.’s in average.

#### 6.2.2. File Size Difference Comparison Using Alaska Image Data Set

File size difference is the difference between the file sizes of the embedded image using the proposed method and Huang et al.’s. This is used to show that the proposed method produces embedded image with smaller file size. Figure 8b summarizes the result for the average file size difference when different payload sizes are embedded. The negative value means that the file size of the embedded image using the proposed method is smaller than Huang et al.’s, and opposite for the positive values. Clearly, the proposed method has smaller file size gain due to embedding in average.

#### 6.2.3. Payload Gain Comparison Using Alaska Image Data Set

Payload gain is the difference between the maximum payload sizes of the Huang et al.’s and the proposed method. This is compared to show the difference in maximum embeddable payload size for each image. Figure 8c summarizes the result. Positive payload gain means that the proposed method can embed that much more, and opposite for the negative values. For all cases except one, the proposed method embeds more than Huang et al.’s for both QF = 50 and QF = 80. In average, proposed method can embed 1570 bits more for QF = 50, and 1163 bits more for QF = 80.

Furthermore, Huang et al.’s could not embed any payload for 24 out of 1000 images for QF = 50, and 35 out of 1000 images for QF = 80, whereas the proposed method manages to embed in all images. The result clearly shows that the proposed method can embed far more than Huang et al.’s.

## 7. Effectiveness of the Block Sorting Algorithm in General

In this section, we analyze and discuss the effect of the block sorting algorithm with respect to the prediction error histogram resulting from the proposed method. In reversible data hiding, block sorting algorithm is used to control the rate of embedding such that the smallest number of **DC** can be used for embedding to minimize the impact on the PSNR.

In the proposed method, only prediction error valued 0 and −1 are used for embedding. However, it can be modified to embed in other prediction error values, such as 1 and −1, or even multiple pairs. Naturally, it is not possible to measure the performance of the block sorting algorithm with respect to all possible modifications.

Instead of measuring the performance for all possible modification, we proposed analyzing the prediction error histogram using the entropy to measure the general effectiveness of the block sorting algorithm. Ideally, block sorting algorithm should sort the blocks such that blocks which are more likely embeddable are prioritized for embedding. In other words, the entropy should increase as more of the less likely embeddable blocks are used for embedding if the block sorting algorithm is well designed.

**Figure 9:**
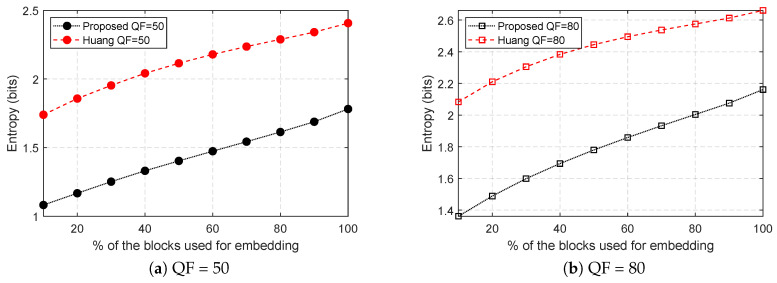
Average prediction error entropy.

Figure 9 shows the average prediction error entropy of 1000 images from Alaska image data set. Each point is measured for every 10 percentage of the total blocks are used for embedding. From the figure, it is very clear that the entropy is smoothly increasing as a greater number of blocks are used for embedding for both methods. Furthermore, the proposed method has much lower average entropy than the Huang et al.’s (DPCM prediction error histogram).

## 8. Conclusions

Reversible data hiding in JPEG image is becoming an important and highly researched topic. With existing work mostly focusing on embedding only using the quantized AC coefficients, there are no good embedding method only using **DC** (quantized DC coefficients). This is a problem for data analytic applications where **AC** (quantized AC coefficients) are used for feature extraction for fine details. The proposed method proposes a novel reversible data hiding in **DC**. It divides the JPEG blocks into two non-overlapping sets and an accurate predictor is proposed, which uses the four neighboring blocks and the **AC**s. The experimental results show that the proposed method performs better than the existing work.

## Abbreviations

The following abbreviations are used in this manuscript:

DCT	Discrete Cosine Transformation
DCT	(Bold font) Quantized DCT coefficients
DC	(Bold font) Quantized DC coefficient
AC	(Bold font) Quantized AC coefficients
PSNR	Peak signal-to-noise ratio
DPCM	JPEG Differential pulse code modulation
QF	Quantization factor
